# Bulleyaconitine A reduces fracture-induced pain and promotes fracture healing in mice

**DOI:** 10.3389/fphar.2023.1046514

**Published:** 2023-01-23

**Authors:** Jun Peng, Sheng Xiao, Juan Xie, Wan Fu

**Affiliations:** Department of Neurology, The First Affiliated Hospital, Hengyang Medical School, University of South China, Hengyang, Hunan, China

**Keywords:** bulleyaconitine A, fracture pain, pain management, fracture healing, analgesic

## Abstract

A fracture is a severe trauma that causes dramatic pain. Appropriate fracture pain management not only improves the patient’s subjective perception, but also increases compliance with rehabilitation training. However, current analgesics for fracture pain are unsatisfactory because of their negative effects on fracture healing or addiction problems. Bulleyaconitine A (BLA), a non-addictive analgesic medicine, is used for the treatment of chronic pain of musculoskeletal disorders in clinical practice, whereas the effects of BLA on fracture pain is undefined. To evaluate the analgesic effects of BLA on fracture, we generated tibial fracture mice here. It is found that oral administration of BLA to mice alleviates fracture-induced mechanical and thermal hyperalgesia. Interestingly, BLA significantly increases locomotor activity levels and reduces anxiety-like behaviors in fractured mice, as determined by open-field test. Notably, BLA treatment promotes bone mineralization and therefore fracture healing in mice, which may be attributed to the increase in mechanical stimulation caused by exercise. Our study suggests that BLA can be used as a promising analgesic agent for the treatment of fracture pain.

## Introduction

Bulleyaconitine A (BLA) is a C-19 diterpene diester alkaloid isolated from *Aconitum bulleyanum* plants in China since 1985. Studies have confirmed the analgesic, anti-inflammatory and anti-anxiety effects of BLA (Xie et al., 2018; Huang et al., 2020). BLA has been approved by China Food and Drug Administration as intramuscular injections, tablets and soft gel capsules for clinical application since 1980s (Weng et al., 2005). BLA tables, an oral medicine, has been prescribed as treatment for rheumatoid arthritis, frozen shoulder and muscle strain for its potent analgesic and anti-inflammatory effects (Wang et al., 2007) (Xie et al., 2018). BLA exerted analgesic effects mainly *via* blocking voltage-gated sodium channels in dorsal root ganglion (DRG) neurons (Xie et al., 2018; Li et al., 2022). Compared with morphine, BLA has the advantage of being non-addictive. It is reported that morphine combined with BLA therapy attenuates morphine tolerance and inhibits morphine-induced withdrawal symptoms in rats (Mai et al., 2020; Zhao et al., 2021), making BLA a novel and safer alternative for clinical analgesia.

Fractures are one of the most common orthopedic injuries and cause severe pain. Among 179 surgical procedures, orthopedic operations are considered to be the most painful experiences, receiving the highest pain score (Gerbershagen et al., 2013). Unlike the repairment of soft tissue, exercise and weight bearing promote fracture healing (Anani and Castillo, 2022). However, rehabilitative training is often inadequate because of unbearable bone pain induced by fracture. Although fracture pain seriously affects the quality of life and rehabilitation of patient, fracture pain management is not well implemented in clinic. Non-steroidal anti-inflammatory drugs (NSAIDs) and opiates are the most commonly used analgesics for the treatment of severe pain following fracture (McVeigh et al., 2020). However, as long-term application of NSAIDs is associated with higher rates of delayed union or non-union, NSAIDs are considered a risk factor for fracture healing (George et al., 2020; Kim et al., 2021). Opiates exhibit powerful analgesic effects, but opioid dependence and their negative effects on osteogenesis limit their clinical use (Coluzzi et al., 2020). Therefore, it is of great significance to investigate novel analgesic agents for the treatment of poorly served fracture pain without adverse effects on fracture healing.

In this present study, we explored the effects of BLA on pain relief and fracture healing in mice fracture models, which offers opportunities for developing novel therapeutic strategies for fracture pain management.

## Materials and methods

### Animals and treatments

Male C57BL/6J mice (3-month-old) were obtained from Hunan SJA Laboratory Animal Company (Hunan, China). The mice were raised in specific pathogen-free (SPF) facility at controlled humidity and temperature, with a 12 h dark/light cycle. The mice were randomly divided into two groups and intragastrically administrated with 0.5 mg/kg/d BLA (MedChemExpress, New Jersey, United States of America) or an equal volume of vehicle from the day before fracture surgery until 4 weeks after fracture. Tibial fracture Surgery was performed as previously described with modifications (Feng et al., 2017). Briefly, mice were anesthetized by inhalation of 2%–4% isoflurane gas. The right hind limb was shaved and disinfected with iodine. An skin incision was made, and the muscles were separated to expose tibial diaphyses. A transversal fracture was generated in the mid-diaphyseal tibia. Then, a 25-gauge syringe needle was inserted into the tibial canal from the tibial plateau for fixation. The muscle and skin were sutured with 5-0 silk. The success of mid-diaphyseal fracture were confirmed by X-ray immediately after surgery. The mice were placed on a 37°C thermal blanket until reviving. All procedures were performed in accordance with the Institutional Animal Ethics Committee and the University of South China Animal Care Guidelines for the Use of Experimental Animals.

### Microcomputed tomography (μCT) analysis

The Mice were euthanatize on day 14, 28 post-fracture respectively. The fractured tibiae were dissected and fixed in 4% paraformaldehyde for 24 h and scanned by vivaCT80 (SCANCO Medical AG, Bruettisellen, Switzerland). The scanning parameters were set at a resolution of 13.8 μm, voltage of 50 kV, a current of 400 μA. To analyze the callus formation, 100 continuous slices (50 slices up and 50 slices down from the breaking line) covering the middle of the newly formed callus were chosen as region of interest (ROI). Bone volume (BV) and bone volume fraction (BV/TV) of callus, excluding the native cortical bone, were calculated by micro-CT software.

### Pain behavior assessments

Three tests were performed to evaluate the fracture-induced pain behaviors: 1) mechanical nociception was assessed by the withdrawal response to von Frey filament stimulation; 2) thermal nociception was assessed by the withdrawal response to thermal stimulation (hot-plate test). 3) Locomotor activity and anxiety-like behaviors in fracture mice were observed by open-field test. Pain-like behaviors were tested 2 h after BLA administration on day 7, 14, 21 and 28 post-fracture. Baselines for these behaviors were assessed 1 day before fracture surgery.

### Mechanical nociception

Mechanical nociception of the fractured hind paws was assessed by von Frey filaments (EXACTA; United States of America) as previously described (Minville et al., 2011). The mice were placed beneath a 10 × 12 × 10 cm clear plastic box with an elevated mesh floor and allowed to acclimate. Paw withdrawal responses to mechanical stimulation were determined by using calibrated von Frey filaments (from 0.02 to 2.56 g) against the paw plantar skin of the fractured hind. The filament was pushed to a slight bend and then held in that position for 6 s. Each von Frey filament was used once starting at 0.02 g and then continued with higher intensity filaments until a withdrawal response was reached, which was considered a positive response. The test was replicated 3 times. The lowest force that produced a response in all three trials was considered the withdrawal threshold.

### Thermal nociception

Thermal nociception was determined by a hot-plate test with modification (Minville et al., 2011). The mice were placed on a hot plate setting a temperature of 50°C. The paw withdrawal latency that mice place their hind paws on the hot plate reflects thermal hyperalgesia. The mice were removed from the plate after a maximal time of 30 s to avoid thermal harm. Final result was the average of three repetitions for each mouse.

### Locomotor activity

Open-field test was performed to observe locomotor activity and anxiety-like behaviors in fracture mice. The test was conducted in a quiet and dimly lit environment. Mice were placed in a 42 × 42 × 42 cm polyvinyl chloride (PVC) box which was divided into a central field (center, 24 × 24 cm) and a peripheral field for analysis purposes. The box was cleaned with 75% ethanol and paper towel before testing each mouse. Spontaneous activities for a 5-min period were recorded with a camera installed above the arena. Behaviors, including the movement distance, movement duration, rearing, frequency of entries to the central area and time spent in the central area were analyzed by EthoVision XT 11.5 software (Noldus Information Technology, United States of America).

### Histochemical staining analysis

The fractured tibiae were dissected and fixed in 4% paraformaldehyde. After dehydration with graded ethanol, the samples were embedded in paraffin and then cut into 5 μm sections. To distinguish newly formed bone and cartilage in the callus, the sections were stained with Safranin-O/fast green (Solarbio Life Sciences, China) according to the manufacturer’s instructions. Tartrate-resistant acid phosphatase (TRAP) staining was performed using a commercial kit (Sigma-Aldrich, United States of America) to determine osteoclastogenesis that would be dyed red. The surface fractions of bone, cartilage and osteoclast in the callus were measured by ImageJ 1.8.0 software.

### Statistical analysis

Behavioral data are presented as mean ± SEM (standard error of the mean). Bone evaluation data are presented as mean ± SD (standard deviation). Statistical analyses of behavioral data were performed using repeated-measures two-way ANOVA with Dunnett’s or Bonferroni’s *post hoc* tests, as indicated in the figure legends. The unpaired, two-tailed Student’s t-test was used to compare means between two groups. Analyses were performed using GraphPad Prism software and the differences were judged to be statistically significant when *p* < 0.05.

## Results

### BLA improves mechanical and thermal hyperalgesia induced by fracture

**FIGURE 1:**
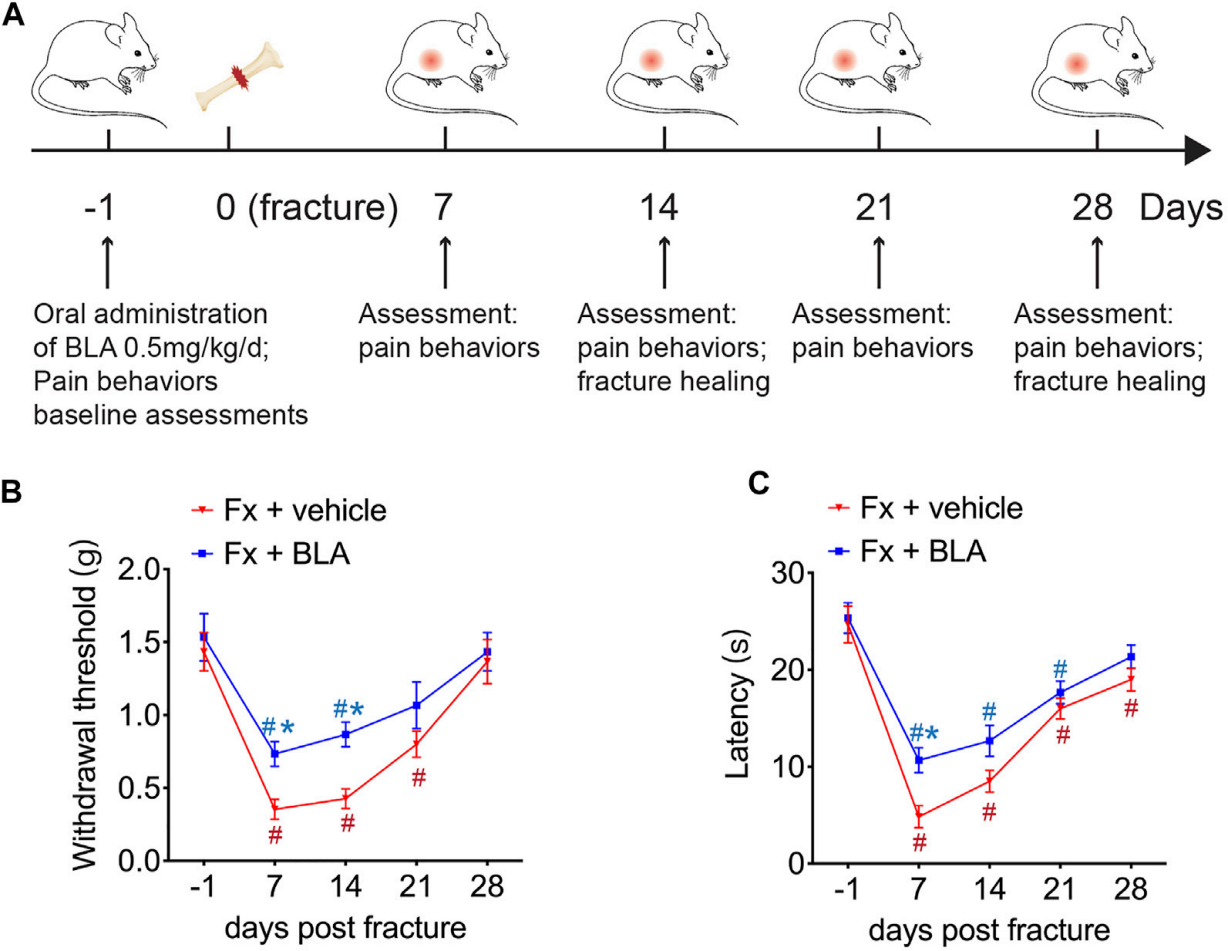
Effects of BLA on mechanical and thermal hyperalgesia after fracture. **(A)** Schematic showing the experimental protocol. **(B)** Paw withdrawal threshold responding to von Frey filaments was tested at the fractured hind paw of BLA or vehicle treated fracture (Fx) mice. *N* = 6 per group. **(C)** Thermal nociceptive withdrawal latency assessing thermal hyperalgesia was tested at the fractured hind paw of BLA or vehicle treated fracture (Fx) mice. *N* = 6 per group. Values are expressed as mean ± SEM. Two-way repeated-measures ANOVA, Dunnett’s multiple-comparisons test for differences within each group: #*p* < 0.05 when compared with baseline (day-1); Bonferroni’s multiple-comparisons test for differences between groups: *p < 0.05 when compared with vehicle group.

To evaluate the analgesic effect of BLA on fracture, we generated tibial fracture mice treated with BLA or vehicle intragastrically. Pain-like behaviors were measured on 1 day before fracture, which served as a baseline, and on day 7, 14, 21 and 28 after the fracture. The establishment of hyperalgesia after fracture is generally employed to assess the degree of fracture induced nociception(Alves et al., 2016; Shi et al., 2021). Mechanical hypersensitivity of the fractured hindlimb was examined by paw withdrawal thresholds response to von Frey filament stimulation. As shown in Figure 1B, fracture induced a significant decline in the mechanical withdrawal thresholds on day 7, 14 and 21post-fracture compared to that of baseline, which did not return until about 28 days after the fracture. However, oral administration of BLA significantly increased the scores of mechanical withdrawal thresholds on day 7 and 14 post-fracture relative to that of fracture mice treated with vehicle. Fracture-induced thermal hyperalgesia was determined by using hot plate test. Similarly, the latencies of thermal withdrawal thresholds were remarkably reduced immediately after fracture that persisted 3-4 weeks, whereas BLA treatment significantly increased the withdrawal latencies by day 7 post-fracture. To determine whether sham operation (mice subjected only to placement of the intramedullary pin without fracture) and BLA administration have effects of on the hyperalgesia in normal mice, we tested the mechanical and thermal withdrawal thresholds in sham-operated and BLA-treated normal mice. However, no obvious changes were found in both sham-operated and BLA-treated normal mice during 14 days (Supplementary Figure 1), indicating that sham operation and BLA treatment have no significant effects on mechanical and thermal hyperalgesia in normal mice. These results suggest that mechanical and thermal hyperalgesia resulting from fracture are alleviated by BLA treatment in mice.

### The effects of BLA on locomotive behaviors of fracture mice

**FIGURE 2:**
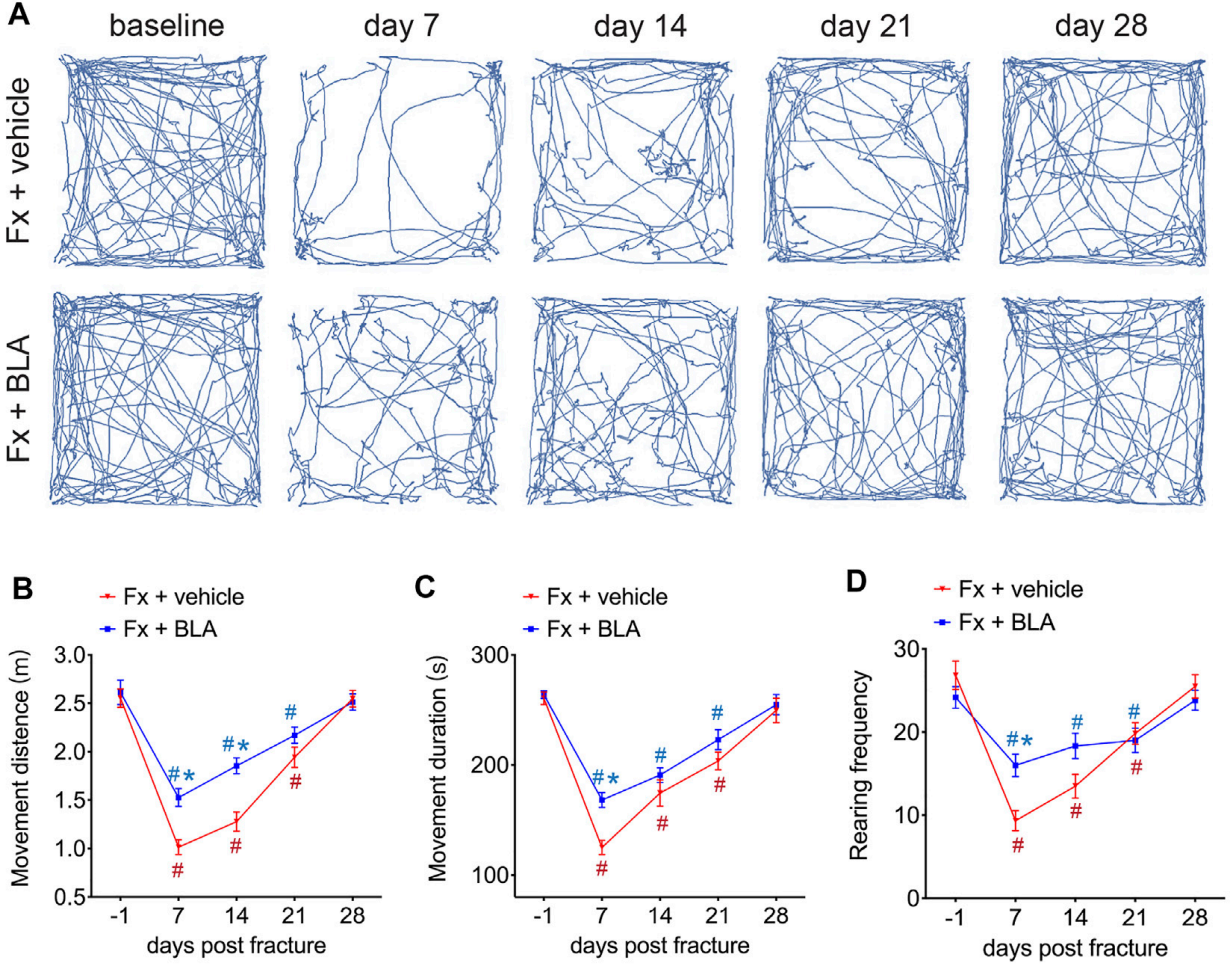
The Effects of BLA on nociceptive behaviors. **(A)** Representative movement traces at serial time points after fracture in mice treated with BLA or vehicle in the open field test. Fx, fracture. **(B)** Quantitative analysis of movement distance of the two groups. *N* = 6 per group. **(C)** Quantitative analysis of movement duration of the two groups. *N* = 6 per group. **(D)** Quantitative analysis of rearing frequency of the two groups. *N* = 6 per group. Values are expressed as mean ± SEM. Two-way repeated-measures ANOVA, Dunnett’s multiple-comparisons test for differences within each group: #*p* < 0.05 when compared with baseline (day-1); Bonferroni’s multiple-comparisons test for differences between groups: *p < 0.05 when compared with vehicle group.

The open-field test, a commonly used experiment for observing locomotive activity in rodents, was performed to observe the change of locomotive behaviors after fracture in mice treated with BLA or vehicle. As shown in Figure 2A, the movement distance was markedly reduced in fracture mice and did not return to normal until about 28 days post-fracture (Figures 2A, B). Moreover, an obvious decrease in the movement duration was also determined in mice during 3 weeks after fracture relative to that of baseline (Figure 2C), indicating that fracture mice had dramatically decreased activity for at least 2–4 weeks after fracture, which is consistent with the time window for pain. Importantly, mice treated with BLA present more activity during 4 weeks post-fracture, as evidenced by obviously increased distance and duration of movement by day 7, 14, 21 after fracture relative to that of fracture mice treated with vehicle (Figures 2A–C). No obvious changes were found in the movement distance and duration in both sham-operated mice and normal mice treated with BLA during 14 days after intervention (Supplementary Figure 2A–C). These results indicating that BLA increases the movement of fracture mice, possibly by alleviating pain after fracture.

Rearing, a common exploratory behavior in mice, increases pressure on the hind paws and may be restrained to avoid causing discomfort in fracture mice. We counted the frequency of rearing and found that rearing frequency is dramatically decreased by day 7, 14, 21 after fracture compared to baseline (Figure 2D). Nevertheless, fracture mice administrated with BLA had significant higher frequency of rearing by day 7 relative to that of vehicle group (Figure 2D). Similarly, no significant changes were found in the rearing frequency in both sham-operated and BLA-treated normal mice (Supplementary Figure 2D). These results indicate that BLA successfully improves the locomotive activity in mice after fracture.

### BLA administration relieves fracture-induced anxiety in mice

**FIGURE 3:**
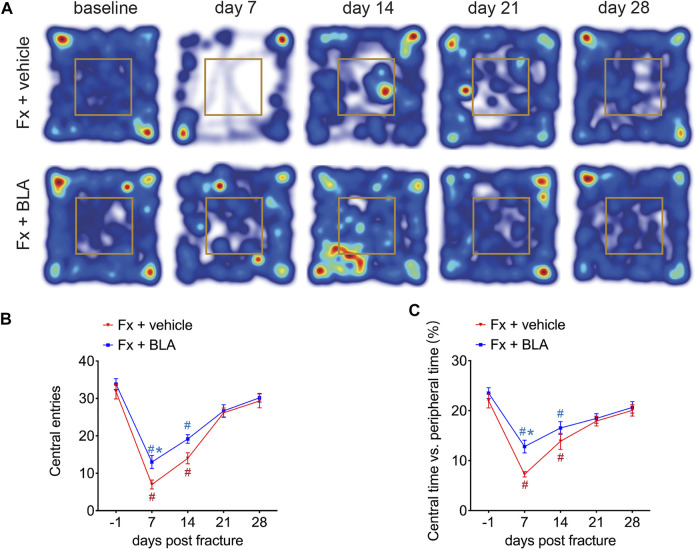
The Effects of BLA on anxiety-like behaviors in fracture mice. **(A)** Representative Heat maps of open-field test comparing movement traces between fracture mice treated with BLA or vehicle. **(B)** Quantitative analysis of entries to the central area of the two groups. *N* = 6 per group. **(C)** Quantitative analysis of time exploring the central area of the two groups. *N* = 6 per group. Values are expressed as mean ± SEM. Two-way repeated-measures ANOVA, Dunnett’s multiple-comparisons test for differences within each group: #*p* < 0.05 when compared with baseline (day-1); Bonferroni’s multiple-comparisons test for differences between groups: *p < 0.05 when compared with vehicle group.

In addition, we assessed anxiety-related behavior in the open-field test. Mice exhibited significantly fewer entries to, and spent less time exploring, the central area by day 7 and 14 after fracture than that of baseline (Figures 3A–C), indicating an increased anxiety of mice during 2 weeks after fracture. Interestingly, mice treated with BLA had significantly increased entries to, and more time spent in, the central area than the vehicle group by day 7 post-fracture (Figures 3A–C). These findings suggest that BLA improves anxiety-like behavior in fracture mice.

### BLA administration facilitates fracture healing

**FIGURE 4:**
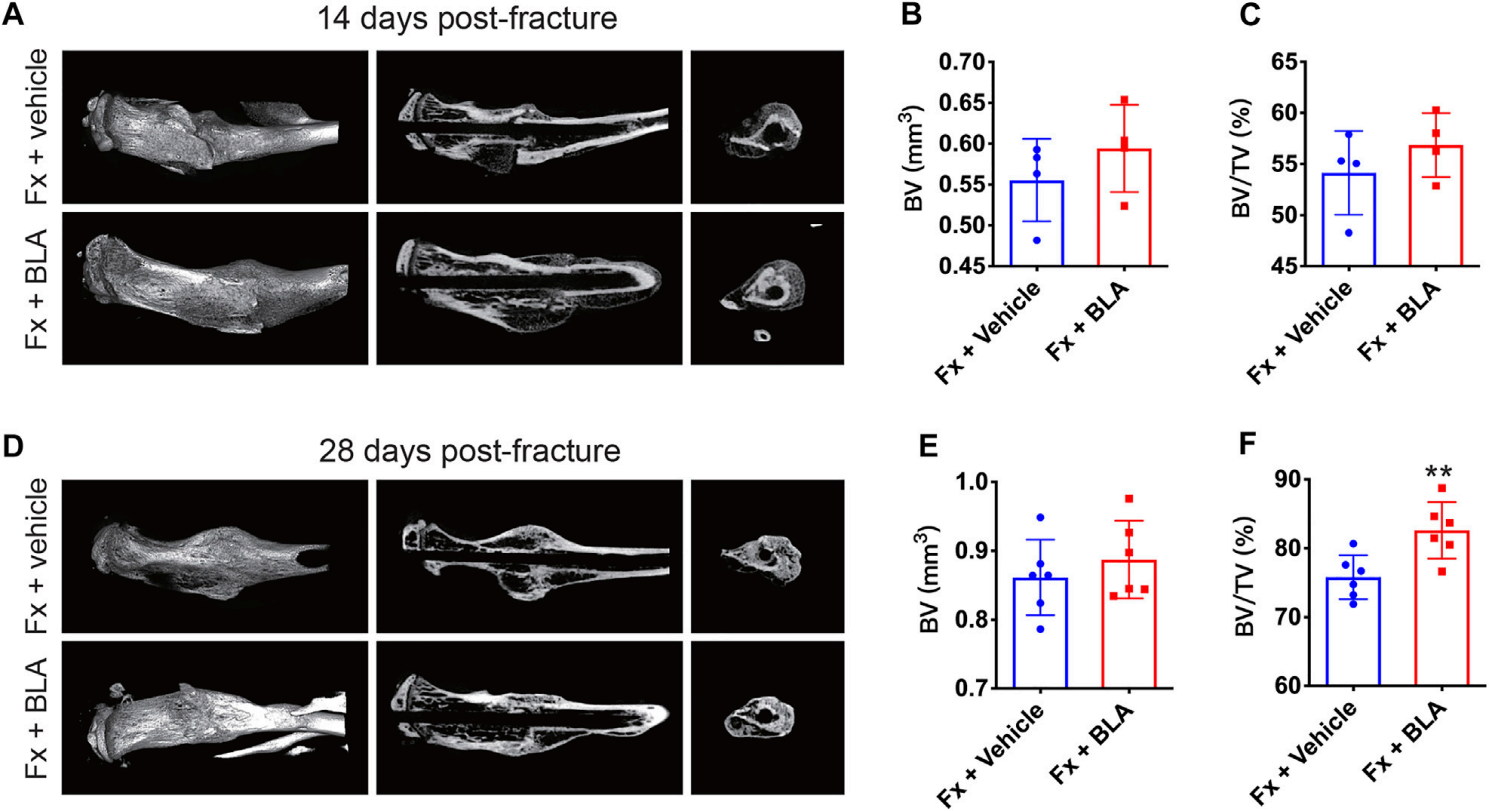
μCT analysis of callus formation **(A)** Representative μCT 3D reconstructions, coronal cross-sectional images, and axial cross-sectional images of fractured tibias from vehicle and BLA groups on day 14 after fracture. **(B, C)** Quantitative analysis of **(B)** the bone volume (BV) and **(C)** the bone volume fraction (BV/TV) of callus on day 14 after fracture. *N* = 4 per group. **(D)** Representative μCT 3D reconstructions, coronal cross-sectional images, and axial cross-sectional images of fractured tibias from vehicle and BLA groups on day 28 after fracture. **(E, F)** Quantitative analysis of **(E)** BV and **(F)** BV/TV of callus on day 28 after fracture. *N* = 6 per group. Values are expressed as mean ± SD. Independent-sample *t*-test: *p < 0.05, **p < 0.01 when compared with vehicle group.

It is reported that BLA suppressed osteoclast formation and bone resorption by inhibiting the NF-κB signal pathway (Zhang et al., 2018). Therefore, we further investigated whether BLA has an impact on fracture healing. Fracture healing can be characterized by three overlapping phases: the inflammatory phase, the repair phase (callus formation and mineralization), and the remodeling phase(Claes et al., 2012). Callus formation and mineralization mainly occur 2–4 weeks after fracture (Li et al., 2019). To assess fracture healing, callus formation by day 14 and 28 after fracture were measured by micro-computed tomography (μCT). Interestingly, 3D μCT reconstructions, coronal cross-sectional and axial cross-sectional images showed that fracture mice treated with BLA had relative more hard callus size than that of mice treated with vehicle by both day 14 post-fracture (Figure 4A), which was confirmed by μCT based quantification of bone volume (BV) and fractional BV to tissue volume (BV/TV) at the site of fracture union (Figures 4B, C). By day 28 after fracture, no obvious difference was observed in the BV of callus between BLA and vehicle groups (Figures 4D, E), whereas a significant increase in BV/TV of callus was determined in mice treated with BLA (Figure 4F), indicating that BLA contributes to accelerated callus remodeling and therefore better fracture healing. These findings suggest that BLA administration promotes fracture healing in mice.

### The effects of BLA on bone mineralization

**FIGURE 5:**
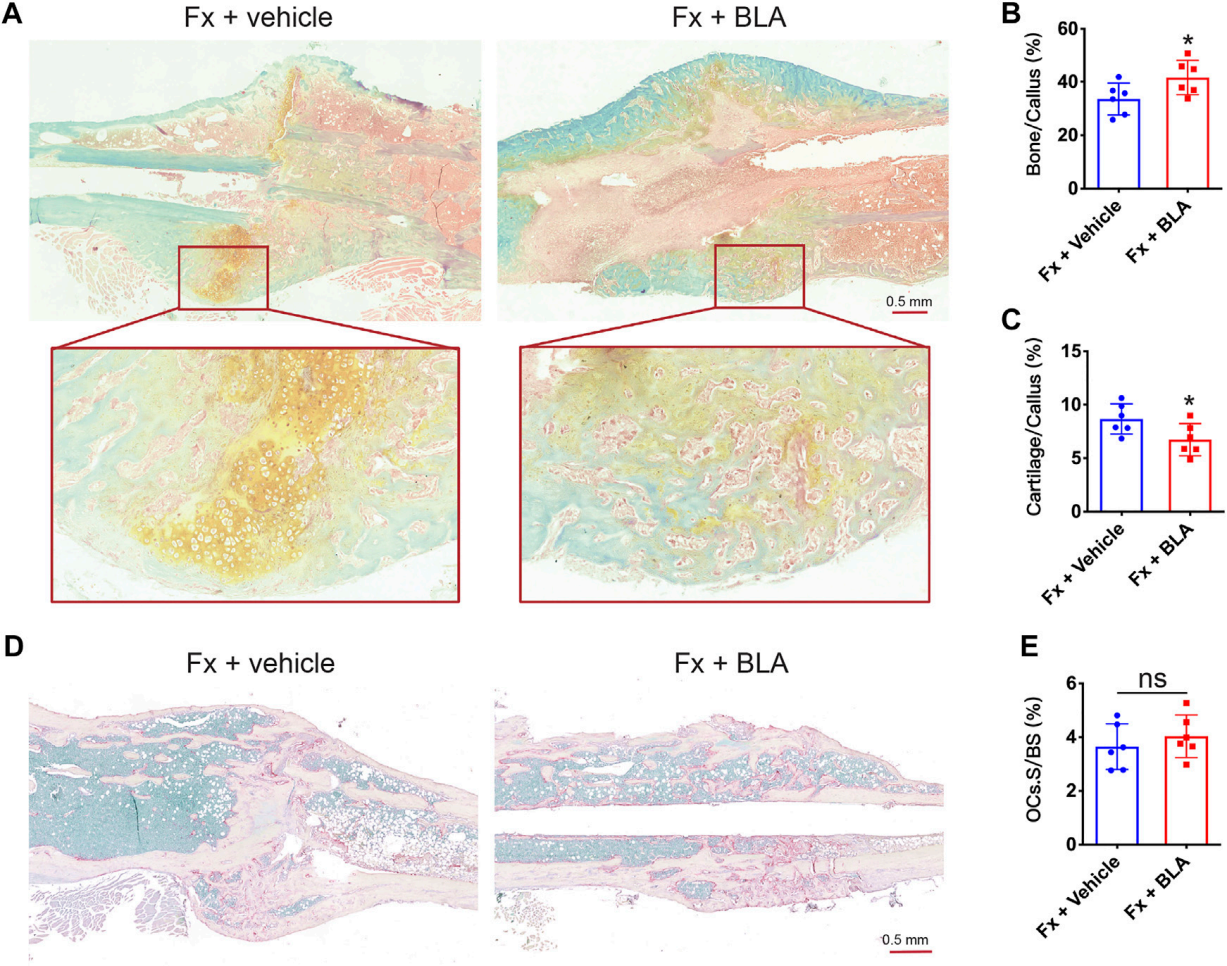
The effects of BLA on bone mineralization **(A)** Representative safranin-O/fast green staining images of fractured tibia sections from vehicle or BLA treated mice on day 28 after fracture. **(B, C)** Quantitative analysis of **(B)** bone fraction and **(C)** cartilage fraction in the callus of different groups. *N* = 6 per group. **(D)** Representative TRAP staining images of fractured tibia sections on day 28 after fracture. **(E)** Quantitative analysis of the fraction of osteoclasts surface (OCs. S) to bone surface (BS). *N* = 6 per group. Values are expressed as mean ± SD. Independent-sample *t*-test: *p < 0.05, when compared with vehicle group.

To assess the level of bone mineralization, we further performed histomorphometry analysis on the sections of the fracture site stained with. Safranin-O/fast green staining showed that fracture mice treated with BLA had more hard callus (green) and less cartilage areas (yellow) of BLA group on day 28 after fracture relative to that of vehicle group (Figure 5A). Quantitative analysis further confirmed a significant increase in hard callus area (Figure 5B) and a decrease in cartilage area (Figure 5C) in BLA treated mice, demonstrating that BLA administration induces faster bone mineralization as well as better fracture healing in mice. Osteoclast formation was evaluated by TRAP staining. However, no significant difference in osteoclast number was observed in the fracture union between BLA and vehicle treated groups on day 28 after fracture (Figures 5D, E). These results indicate that BLA administration promotes bone mineralization of callus in fracture mice.

## Discussion

The number of fractures is expected to increase in the coming years due to the accumulation of osteoporosis caused by the ageing of the population (Compston et al., 2019; Zhang et al., 2020). Fractures often give rise to severe pain, which is a main symptom that requires treatment. An effective fracture pain management not only improves subjective feeling of patients, but also is critical to ensure proper bone healing (Bove et al., 2009). BLA, an oral analgesic agent, is widely used in controlling chronic musculoskeletal pain in clinical practice. In this study, we first examined the effects of BLA on fracture pain and fracture healing. We found that oral administration of BLA significantly relieved pain symptoms and promoted fracture healing in mice, suggesting that BLA is a promising analgesic for fracture pain management.

Studies have reported the establishment of mechanical and thermal hyperalgesia in fracture mice (Magnusdottir et al., 2021), and the paw withdrawal behaviors response to mechanical and thermal stimulations are widely utilized to evaluate fracture pain in animal models (McVeigh et al., 2020). In this study, we also confirmed that mice experienced persistent mechanical and thermal hyperalgesia in 3-4 weeks after tibial fracture, and BLA treatment significantly increased the paw withdrawal thresholds of fractured hindlimbs stimulated by Von Frey fibers or heat. Our results demonstrate that BLA is useful in alleviating both the mechanical and thermal hyperalgesia induced by fracture.

Pain behaviors reflect the changes in quality of life and physical function caused by pain (Gregory et al., 2013; McVeigh et al., 2020), which is closely related to clinical practice and is meaningful for the screening of analgesic drugs. In addition to evoked pain behaviors, locomotive behaviors were also examined to fully assess nociception after fracture. Locomotive activity and rearing, examined by open field test, are commonly used in the assessment of fracture pain (Mitchell et al., 2018; McVeigh et al., 2020). In this study, significant reductions in the distance and duration of movements were observed in fracture mice, which did not returned to normal until 4 weeks after fracture. Decreased movements in mice after fracture may be attributed to pain as well as impaired motor function of the fractured limb. However, the movement distance and duration of fracture mice increased by about 30% after the BLA intervention, suggesting that fracture mice treated with BLA may have less pain feeling during normal exercise and more daily activities. Rearing, an exploratory behavior increasing weight bearing upon the hindlimbs, was rejected by mice immediately after fracture that persists for 1-2 weeks, whereas BLA increased rearing behaviors in mice on day 14 after fracture. Our results indicate that BLA improves locomotor behaviors in fracture mice, which may be attributed to the pain-relieving effect of BLA.

Additionally, we also found that fracture mice exhibited anxiety-like behavior in the open field test and BLA administration improved the anxiety-like behavior of fracture mice. In consistent with our study, it is reported that BLA exerted marked anti-visceral pain and anti-anxiety effects in visceral pain rats (Huang et al., 2020). There exists a potential link between visceral pain and psychological disorders (such as anxiety and depression). The anti-anxiety effect of BLA may result from pain relief.

Currently, opioids and NSAIDs are the two primary drugs prescribed to manage pain for fracture and post-surgical pain. However, the negative impact on fracture healing and the development of tolerance and dependence make them unsatisfactory in clinical practice (George et al., 2020)**.** Interestingly, BLA administration not only improves fracture-induced pain symptoms, but also facilitates fracture healing in mice, as evidenced by increased bone calluses and faster bone mineralization. It is well known that mechanical loading is beneficial to bone formation, which is also an important reason for rehabilitation training to promote fracture healing. Here, we think that the significant increase in movements, which enhances mechanical stimulation, may account for the better fracture healing observed in BLA treated mice. Zhang et al. have reported that BLA prevents Ti particle-induced osteolysis *via* suppressing osteoclastogenesis and promoting osteoblastogenesis (Zhang et al., 2018). Nevertheless, no significant difference was observed in the number of osteoclasts at the fracture site in this study, which may attribute to the difference in the drug delivery methods and animal models. The osteogenic effect of BLA on bone metabolism is interesting, which should be investigated in the future studies.

BLA exerts analgesic effects by multiple mechanisms including the blockade on Nav channels in DRG neurons, the inhibition of long-term potentiation at C-fiber synapses in spinal dorsal horn and the modulation of microglial functions (Li et al., 2022). However, we have not verified the molecular mechanism by which BLA alleviates fracture pain in this study. In addition, the influence of BLA on later fracture healing (callus remodeling period) has not been observed. Much research remains to be done to fully understand the therapeutic effects of BLA on fracture pain and bone metabolism before clinical application.

BLA has been listed in the Pharmacopoeia of the People’s Republic of China (2015) (Pharmacopoeia of China, 2015) and applied in clinical treatment for rheumatoid arthritis, osteoarthritis and chronic musculoskeletal pain disorders, receiving good curative effects. BLA, which is not a NSAID, exerts its effects by modulating sodium ion channels. Therefore, BLA has few NSAID and opioid-related adverse effects, such as gastrointestinal, cardiovascular, renal toxicity and psychological dependency. Rarely, patients may experience temporary mild palpitations, nausea, numbness of the lips and tongue, and palpitations after taking the drug, according to the drug’s instructions.

In conclusion, oral administration of BLA is able to improve fracture-induced mechanical and thermal hyperalgesia, anxiety-like behaviors and motor activity. Furthermore, increased callus formation and accelerated bone mineralization were observed in fracture mice treated with BLA. Our findings suggest that BLA may be a novel and potential analgesic for fracture pain management.

## Data Availability

The original contributions presented in the study are included in the article/Supplementary Material, further inquiries can be directed to the corresponding author.
