# Supplementary material for: Bulleyaconitine A reduces fracture-induced pain and promotes fracture healing in mice
Source: Front Pharmacol. 2023 Jan 23;14:1046514. doi: 10.3389/fphar.2023.1046514 (PMC9899823; doi:10.3389/fphar.2023.1046514)
Supplement: Supplementary file 1 [file DataSheet1.PDF]

Supplementary material

Bulleyaconitine A reduces fracture-induced pain and promotes fracture healing in mice

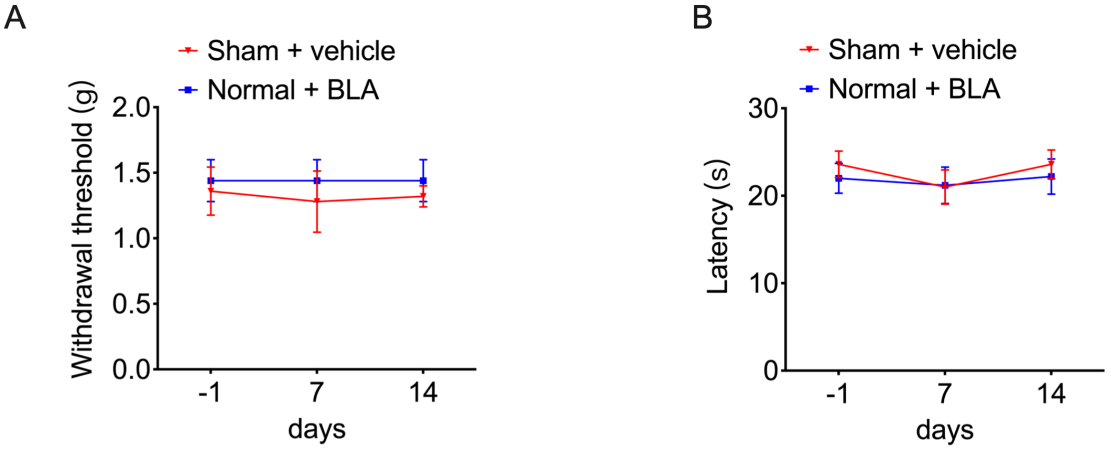

Supplementary figure 1. Effects of sham operation without fracture and BLA on mechanical and thermal hyperalgesia in normal mice. (A) Paw withdrawal threshold responding to von Frey filaments was tested at the hind paw of sham-operate and BLA-treated mice. N=5 per group. (B) Thermal nociceptive withdrawal latency assessing thermal hyperalgesia was tested at hind paw of different groups. N=5 per group. Values are expressed as mean ± SEM. Two-way repeated-measures ANOVA, Dunnett's multiple-comparisons test for differences within each group; Bonferroni's multiple-comparisons test for differences between groups.

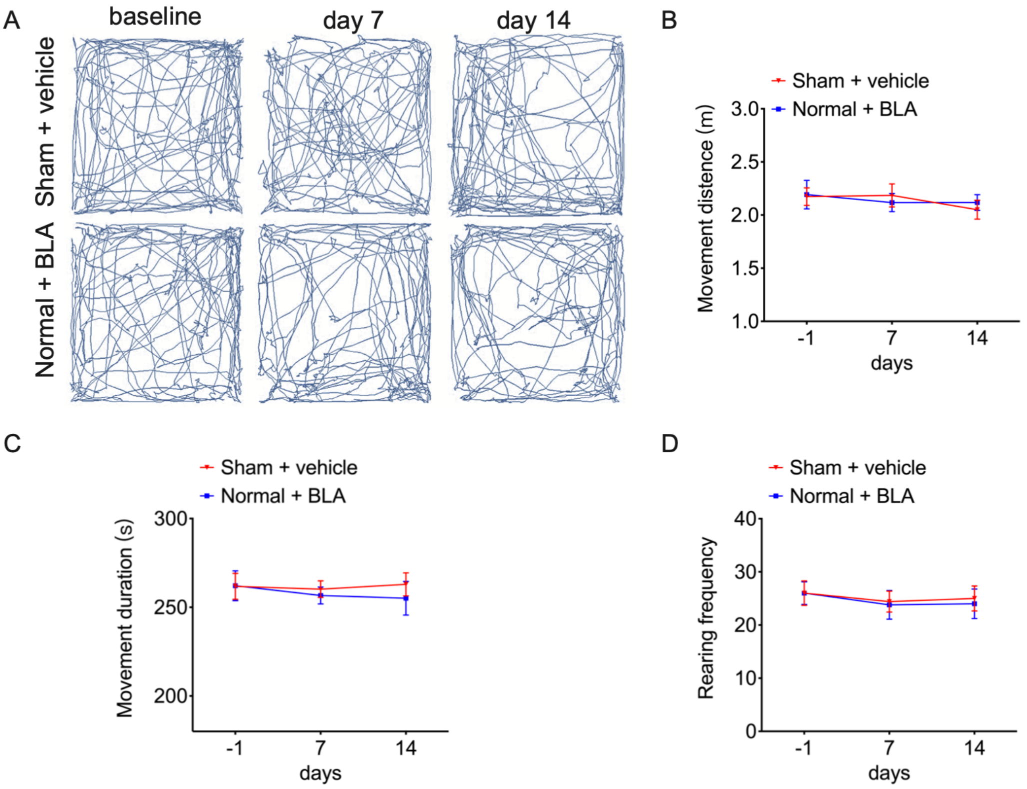

Supplementary Figure 2. The Effects of sham operation without fracture and BLA on nociceptive behaviors. (A) Representative movement traces at serial time points in sham-operate or BLA-treated normal mice in the open field test. (B) Quantitative analysis of movement distance of the two groups. N=5 per group. (C) Quantitative analysis of movement duration of the two groups. N=5 per group. (D) Quantitative analysis of rearing frequency of the two groups. N=5 per group. Values are expressed as mean ± SEM. Two-way repeated-measures ANOVA, Dunnett's multiple-comparisons test for differences within each group; Bonferroni's multiple-comparisons test for differences between groups.
